# Design of Liposomes Carrying HelixComplex Snail Mucus: Preliminary Studies

**DOI:** 10.3390/molecules26164709

**Published:** 2021-08-04

**Authors:** Andrea Alogna, Valentina Gentili, Claudio Trapella, Supandeep Singh Hallan, Maddalena Sguizzato, Giovanni Strazzabosco, Mercedes Fernández, Rita Cortesi, Roberta Rizzo, Daria Bortolotti

**Affiliations:** 1 Department of Chemical, Pharmaceutical and Agricultural Sciences, University of Ferrara, I-44121 Ferrara, Italy; gntvnt@unife.it (V.G.); trap@unife.it (C.T.); hllsnd@unife.it (S.S.H.); sgzmdl@unife.it (M.S.); giovanni.strazzabosco@edu.unife.it (G.S.); mercedes.fernandez@unife.it (M.F.); rbr@unife.it (R.R.); brtdra@unife.it (D.B.); 2 Laboratory for Technologies of Advanced Therapies (LTTA), University of Ferrara, 70 Eliporto Street, I-44121 Ferrara, Italy; 3 Biotechnology Interuniversity Consortium (C.I.B.), Ferrara Section, University of Ferrara, I-44121 Ferrara, Italy

**Keywords:** slime mucus, HelixComplex, liposomes, MTT test, drug delivery

## Abstract

In recent decades liposomes have been used in different field thanks to their ability to act as a vehicle for a wide range of biomolecules, their great versatility and their easy production. The aim of this study was to evaluate liposomes as a vehicle for the actives present in the HelixComplex (HC) snail mucus for topical delivery. Liposomes composed of a mixture of phosphatidylcholine, cholesterol and octadecylamine were prepared with and without HC (empty liposomes) and their biological efficacy was tested by evaluating cell viability and migration. HC-loaded liposomes (LHC) were stable throughout 60 days of observation, and showed interesting effects on wound healing reconstitution. In particular, we observed that 25 µg/mL LHC were already able to induce a higher cell monolayer reconstitution in comparison to the untreated samples and HC treated samples after only 4 h (28% versus 10% and 7%, *p* = 0.03 and *p*= 0.003, respectively). The effect was more evident at 24 h in comparison with the untreated control (54% versus 21.2% and 41.6%, *p* = 0.006 and *p* = NS, respectively). These results represent a preliminary, but promising, novelty in the delivery strategy of the actives present in the HelixComplex mucus.

## 1. Introduction

Since the discovery of lipid vesicles derived from self-forming enclosed lipid bilayers upon hydration, liposome drug delivery systems have played a significant role in the formulation of drugs to improve therapeutics [1]. In fact, lipid vesicles are able to easily interact with cellular membranes and to leave the active bodies directly inside the cells. The liposomes used in these formulations can be either positively or negatively charged, meaning that they will interact differently with organic structures, and can be designed to carry specific signaling molecules on the external surfaces, or inside the double-layer. Because of these abilities, liposomes have the advantage of using low amounts of the active bodies, avoiding toxic effects or excessive price compared to higher concentrations. For this reason, lipid vesicles are used today in a wide range of applications, from the nutraceutical to the pharmaceutical field [2,3,4,5,6,7]. Although several studies have recently reported the use of liposomes in a safe and efficient manner for transdermal systemic folate delivery through the skin barrier [8], the use of lipid vesicles in cosmetic applications as a snail mucus delivery system represents a novelty. 

The snail secretion, or snail mucus, is a mucous substance that covers the entire external surface of the animal and is secreted by particular salivary epidermal glands located at the level of the snail’s foot (pedal glands). The mucus has different functions in the life of the animal, having adhesive, emollient, moisturizing, lubricating, protective and even reparative properties. For these reasons, its use has been proposed for the formulation of para-pharmaceutic products for the management of wounds and for the treatment of chronic bronchitis. HelixComplex Snail mucus (HC) is a raw material produced by the *Helix aspersa* species. This snail extract is characterized by the absence of cellular toxicity and protects cells from apoptosis, as demonstrated by the lower mortality reported in human fibroblast cells culture treated with HC in the presence of low serum concentrations (0.1%), compared to untreated cells [9]. That evidence was confirmed by the absence of cell viability reduction in the presence of increasing concentrations of HC [9]. 

Moreover, HC treatment on human fibroblasts induced significant morphological changes of fibroblasts associated with the reorganization of the cytoskeleton, cell-sized magnification and a positive effect on cell motility, resulting in increased cell migration and tissue repair rate [10]. 

More recently, HC was shown to protect from damage caused by ozone. Ozone is considered one of the most toxic pollutants, causing 21,000 premature deaths each year in Europe in areas exceeding the limits set (70 μg/m^3^). The damage occurs mainly at the level of the lower respiratory tract, but the skin, which is exposed to air pollutants, can also be affected. Ozone reacts with the lipids of the stratum corneum to generate H_2_O_2_, which induces toxicity because of its strong oxidizing power. HC reduces the damage generated by ozone thanks to its protective properties [9]. The presence of mucopolysaccharides makes the secretion a promoter of hydrogen bonds with water, thus increasing the level of hydration when applied to the skin. In addition, these compounds can induce the endogenous synthesis of hyaluronic acid with a consequent increase in the elastic and moisturizing characteristics of the skin. Moreover, HC is able to reduce the inflammatory state observed after ozone exposure of skin, decreasing the levels of inflammatory cytokines (IL-8, IL-6, IL-1beta). 

To date, despite the evidence of snail mucus’ properties and the efficacy of liposome-based formulation in improving actives’ vehiculation, the possibility of using liposomes to enhance snail mucus biological activities has not been explored.

The aim of this study was to understand if HC efficacy might be improved with an efficient drug delivery system based on lipid carriers. For this reason, we produced liposomes composed of phosphatidyl choline (PC), cholesterol (CH) and the charged surfactant octadecylamine (OD) characterized by a high compatibility with aqueous solutions and simplicity of production. Liposomes, produced by direct hydration, were loaded with HC (LHC). LHC were characterized in terms of size, charge, morphology and in vitro activity.

## 2. Materials and Methods

### 2.1. Materials

Soybean lecithin (PC) (90% phosphatidylcholine) used for liposome preparation was Epikuron 200 from Lucas Meyer, Hamburg, Germany. CH and OD were purchased from Sigma-Aldrich (St. Louis, MO, USA). Solvents and all other chemicals were of analytical grade and were from Merck Serono S.p.A. (Rome, Italy). All other materials and solvents of high purity grade were from Sigma Chemical Co. Crystal violet, acetic acid and Methanol from Sigma-Aldrich (St Louis, MO, USA). HelixComplex Snail Mucus was purchased from HelixPharma srl Company (Ferrara, Italy). 

### 2.2. Characteristics of the Snail Mucus

The snail mucus HelixComplex (HC) was produced using a controlled and patented supply chain (Patent number FE102017000117547) and a patented method for the extraction (Patent number BO2011A000590) and filtration (0.2 μm; PALL, Italy). The characterization of HC was performed as previously reported [10]. Briefly, HC was analyzed by IR-spectroscopy, focusing on some specific areas of the spectrum, and characteristics of the product that prove HC standard quality properties, such as: (i) the absorbance peak at 3250 cm^−1^ typical of hydroxyl groups of hydrophilic amino acids; (ii) the area of aromatic overtone between 3000 and 3200 cm^−1^, due to aromatic amino acids; (iii) the most important peaks at 1645 cm^−1^ and at 1540 cm^−1^ typical of amide bond, which indicate the presence of proteins. The absence of bacterial and fungal colonies was tested as previously reported [10].

### 2.3. Liposome Preparation

Taking into account the negative charge of the HC, cationic liposomes were selected to facilitate their electrostatic interaction with the HC protein content. PC/CH/OD (2:1:1 molar ratio) liposomes (L) were prepared by direct hydration method adapted from previously described studies [11,12]. Briefly, 50 mg of lipid phase were dissolved in methylene chloride/methanol mixture (1:1, *v/v*). Afterwards, the organic solvent was removed using a rotary evaporator under vacuum attaining a thin lipid film. The film was then subjected to hydration using 2 mL of HC solution 20 mg/mL, followed by 30 min swirling and a very brief ultrasonication (3 min) in order to avoid HC denaturation. Indeed, as shown by results reported in Figure S1, the ultrasonication process did not affect the biological activity of HC. 

Finally, the liposomal suspension was diluted 1:1 with MilliQ water to reduce aggregation obtaining a final colloidal solution of liposome-carrying HC (LHC) reaching a final HC content of 10 mg/mL.

### 2.4. Liposome Characterization 

Concerning freeze–fracture microscopy, LCH dispersion was sandwiched between copper plates, frozen using liquid propane (−180 °C), transferred to a Balzers BAF 400 freeze replica apparatus (Balzers, Liechtenstein) and subjected to fracture at −150 °C. Samples were immediately replicated with Pt/C (2 nm) at an angle of 45° followed by C (20 nm) at an angle of 90°. After cleaning stripped replicas with 30% sodium hypochloric and potassium dichromate-H_2_SO_4_ solution followed by distilled water, they were mounted on 300-mesh Ni grids, and visualized after drying by means of a transmission electron microscope (JEM200 CX, JEOL, Tokyo, Japan). Micrographs were taken randomly within replica regions representative of the sample.

Vesicle size were measured on aqueous diluted liposome samples (1:20 by volume) by means of photon correlation spectroscopy (PCS) using a Zetasizer Nano S90 (Malvern Instr., Malvern, UK) equipped with a 5 mW helium neon laser with a wavelength output of 633 nm. Measurements were made at 25 °C at an angle of 90°, run time around 180 s. Data were interpreted by the “CONTIN” [13]. 

ζ potential measurements were conducted on Zetasizer ultra (Malvern Ltd., Malvern, UK) using plastic-ware cleaned with detergent and rinsed twice with milliQ water. 

HC encapsulation efficacy into liposomes was determined evaluating the content of allantoin, a characteristic snail mucus component, as reference molecule. Briefly, crude HC was used to prepare LHC dispersion. Afterwards, LHC was loaded on Amicon^®^Ultra Ultracel^®^-100K (100 kDa cut off) centrifugal filter and centrifuged at 5000 rpm for 5 min to separate LHC from residual crude HC. The amount of allantoin in crude HC and filtered LHC was characterized by IR spectra analysis using a FT-IR Perkin-Elmer Spectrum 100 with ZnSe ATR diamond (serial number 40,326) (Waltham, MA, USA). The concentration of allantoin in LHC was determined by subtracting the amount of allantoin found in the filtered LHC from the allantoin concentration of the crude HC. Analyses were performed by HPLC using a Beckman System Gold 125 coupled with a Beckman Coulter System 168 at 220 nm (Brea, CA, USA) with analytical column Synergi 4 μm Hydro-RPA (250 × 4.6 mm) and a standard curve ranging from 0.222 to 0.0138 mg/mL.

### 2.5. Biological Properties Evaluation: Cell Viability and Migration

The effect on cell viability of the liposomes used for encapsulation was evaluated on human fibroblast MRC-5 (ATCC^®^ CCL-171™) by MTT assay (Roche), following product instructions. Cells were treated for 24 h with different concentrations of HC, L or LHC and the viability was assessed by MTT assay (Roche). The HC 2000 and 10,000 µg/mL was used as positive control, as previously reported [9,10]. The regenerative properties of the LHC were evaluated by scratch test assay [14], in comparison with the HC snail mucus. Briefly, the human fibroblast cell line MRC-5 was cultured in 24 wells until the formation of a cellular monolayer and then an artificial scratch was performed with a pipette tip on the culture surface. The cells were then treated with 2 mg/mL of HC Snail Mucus, water for the untreated control or LHC and the percentage of cellular migration has been evaluated after 4 h and 24 h of incubation at 37 °C.

### 2.6. Statistical Analysis

Data obtained from at least three independent experiments were analyzed using the GraphPad Prism v9 software. Results were reported as mean ± SD and analyzed by Student’s *t* test. Results were considered significant when a *p*-value < 0.05 was obtained.

## 3. Results and Discussion

### 3.1. HC Characterization

The main characteristics of HC are summarized in Table 1. 

The main macromolecules of the HC resulted in proteins and glycosaminoglycans, with a small amount of sugars and polyphenols and other secondary metabolites. Their quantification was performed by colorimetric assays and HPLC analysis, i.e., for glycolic acid and allantoin [15,16]. To understand the effect of HC it is necessary to refer to its unique chemical composition. The most consistent chemical species are proteins, with a value between 100 mg/L and 250 mg/L, as characterized by IR spectroscopy (infrared spectroscopy).

The HC IR spectrum contains peaks generated by the presence of hydroxyl groups typical of hydrophilic amino acids (3250 cm^−1^), of aromatic ones (3000 cm^−1^ and 3200 cm^−1)^ and amide bonds, characteristic of the proteins (1645 cm^−1^ and 1540 cm^−1^) (see Results section). 

Other compounds found within HC are: minerals (250–350 mg/L), sugars (0.010–0.027 g/L), collagen (1–100 mg/L) and polyphenols (70–80 mg/L). 

### 3.2. Liposome Preparation and Characterization

LHC composed of PC, CH and OD in mixture 2:1:1 (molar ratio) were prepared by direct hydration method followed by swirling and a very brief ultrasonication in order to avoid HC denaturation.

**Figure 1:**
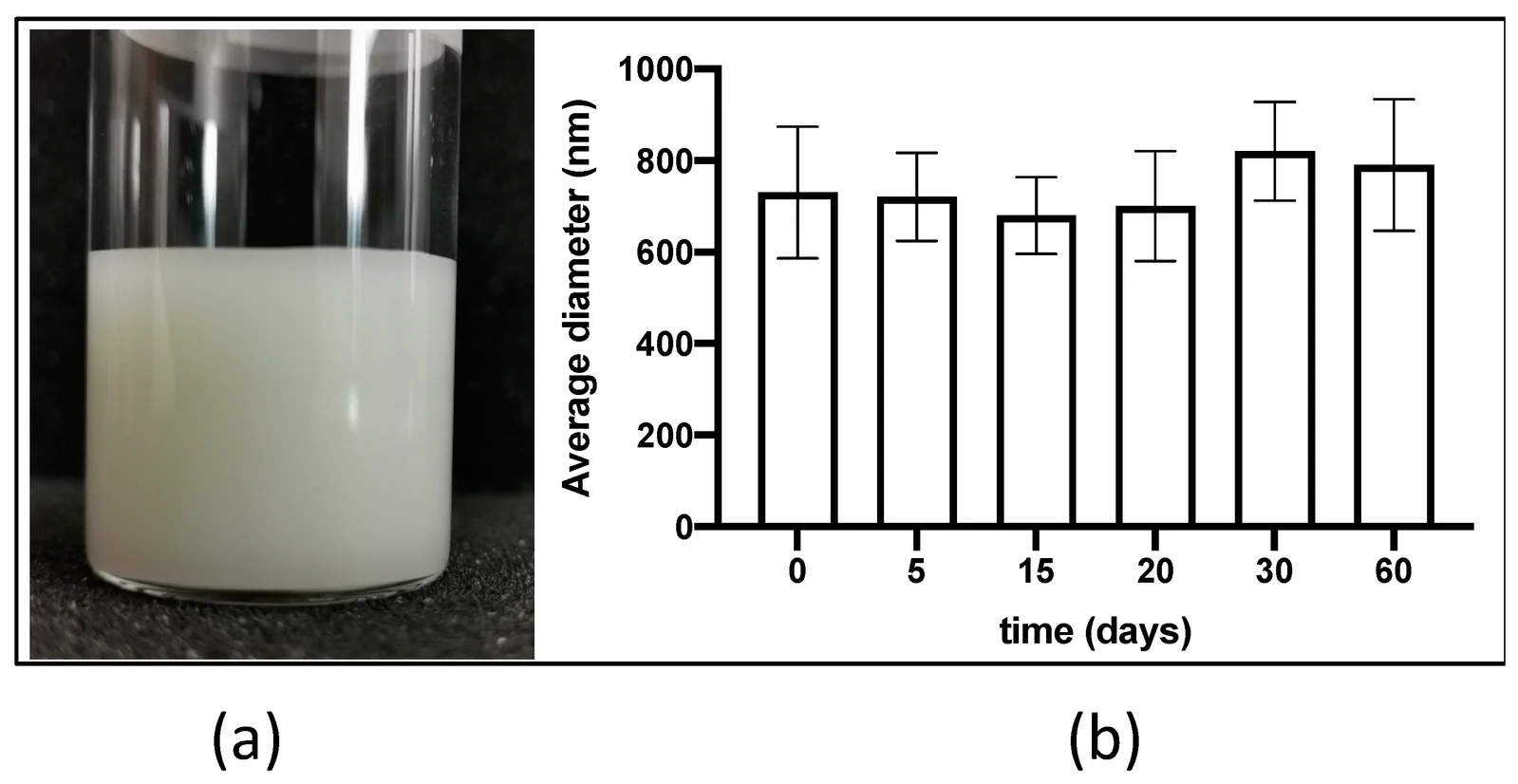
Dispersion macroscopic appearance (**a**) and mean size during time (**b**) of LHC. Data are the mean of five independent determinations on different aliquots of the dispersion ± SD.

We selected liposomes containing OD, namely stearylamine, an established cationic lipid molecule employed in the production of liposomes, as it is often used to regulate the charge of liposomes, therefore increasing the stability of the formulation not only with regard to vesicles’ electrostatic repulsion but also to its preservative activity. In the literature, liposomes containing OD are proposed to carry specific molecules, such as targeting ligands (peptides) and/or polymers (e.g., PEG) demonstrating a strong affinity and interaction with the outer surface of cells [17]. In this view we thought that the use of such a cationic component could be interesting to investigate and proposed a topical application on injured or damaged skin. The LHC formulation showed a macroscopic uniform milky aspect (Figure 1a) and an encapsulation efficacy of HC, indirectly obtained by measuring within the filtered fraction the content of the characteristic snail mucus component allantoin, of 93.39% ± 1.25 (Figure S2). On the basis of the encapsulation efficacy, we calculated the amount of HC present inside the liposomes. Unloaded liposomes (L) and LHC were used at the scalar concentrations reported in Table 2 for biological tests.

During two months of storage at room temperature a good stability of the liposomes in terms of both agglomerate formation (being completely absent) and vesicles’ mean size was observed. Figure 1b reports the average diameter (z-ave) of LHC during 60 days of observation. Possibly, the increased z-ave observed after 30 and 60 days might be ascribed to a partial fusion of liposome vesicles, even though no significant difference was observed as compared to day 0 (*p* = 0.149, Student’s *t* test). 

Taken together the morphological results corroborated the size data obtained by PCS analyses performed on the same day. Indeed, the mean of five independent determinations on different batches of the same LHC dispersion indicated an average diameter (z-ave) of 664.6 ± 58.26 nm and a polydispersity index of 0.135 ± 0.26.

Moreover, LHC showed a ζ potential of +37.67 mV that might physically stabilize this preparation over time due to the surface charge repulsion ascribed to the presence of a cationic surfactant within the phospholipid bilayer.

**Figure 2:**
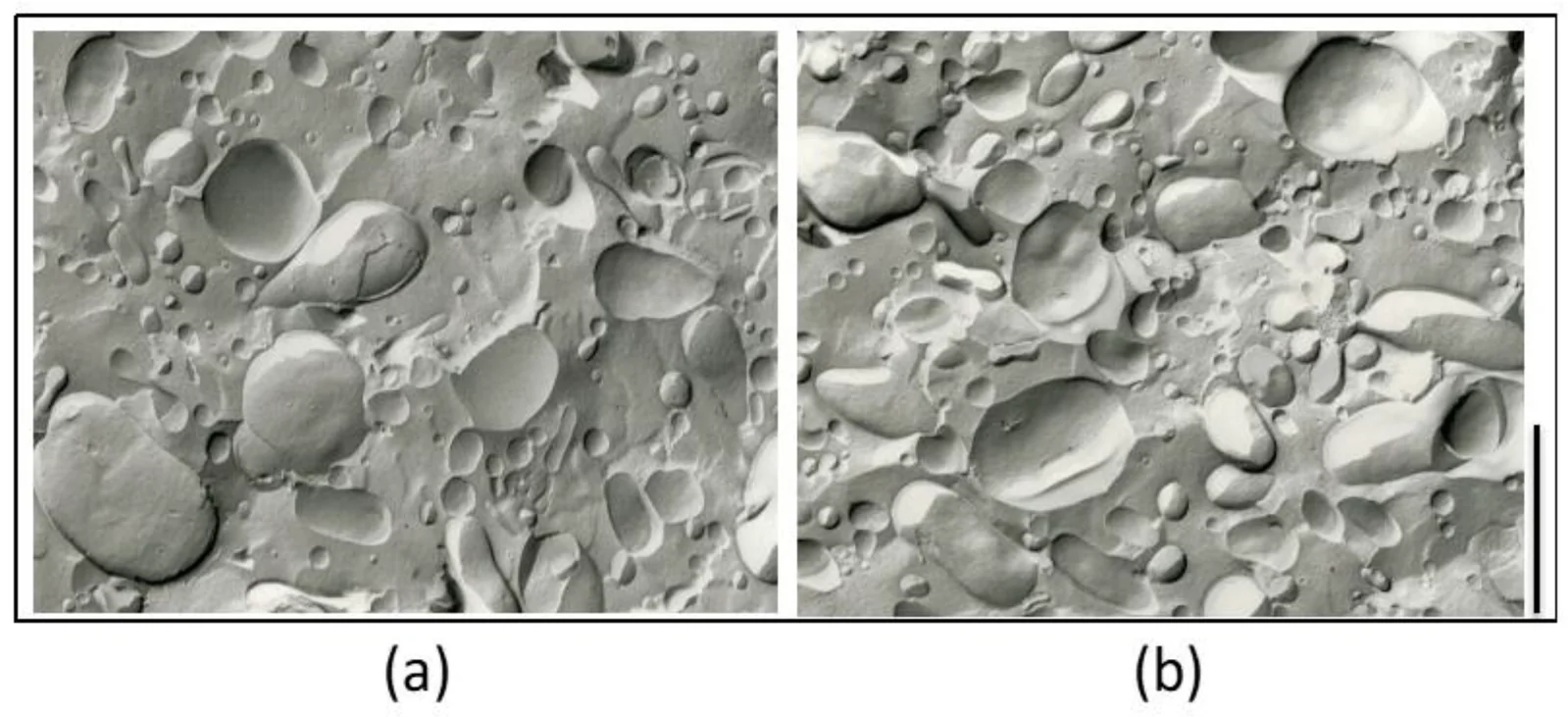
Freeze–fracture electron microphotographs of L (**a**) and LHC (**b**) visualized one day after production. Bar corresponds to 1.0 um.

On the other hand, the addition of HC does not affect the morphology of liposomes, as indicated by freeze–fracture electron micrographs reported in Figure 2, in which unilamellar populations with a certain grade of polydispersity are detectable.

**Figure 3:**
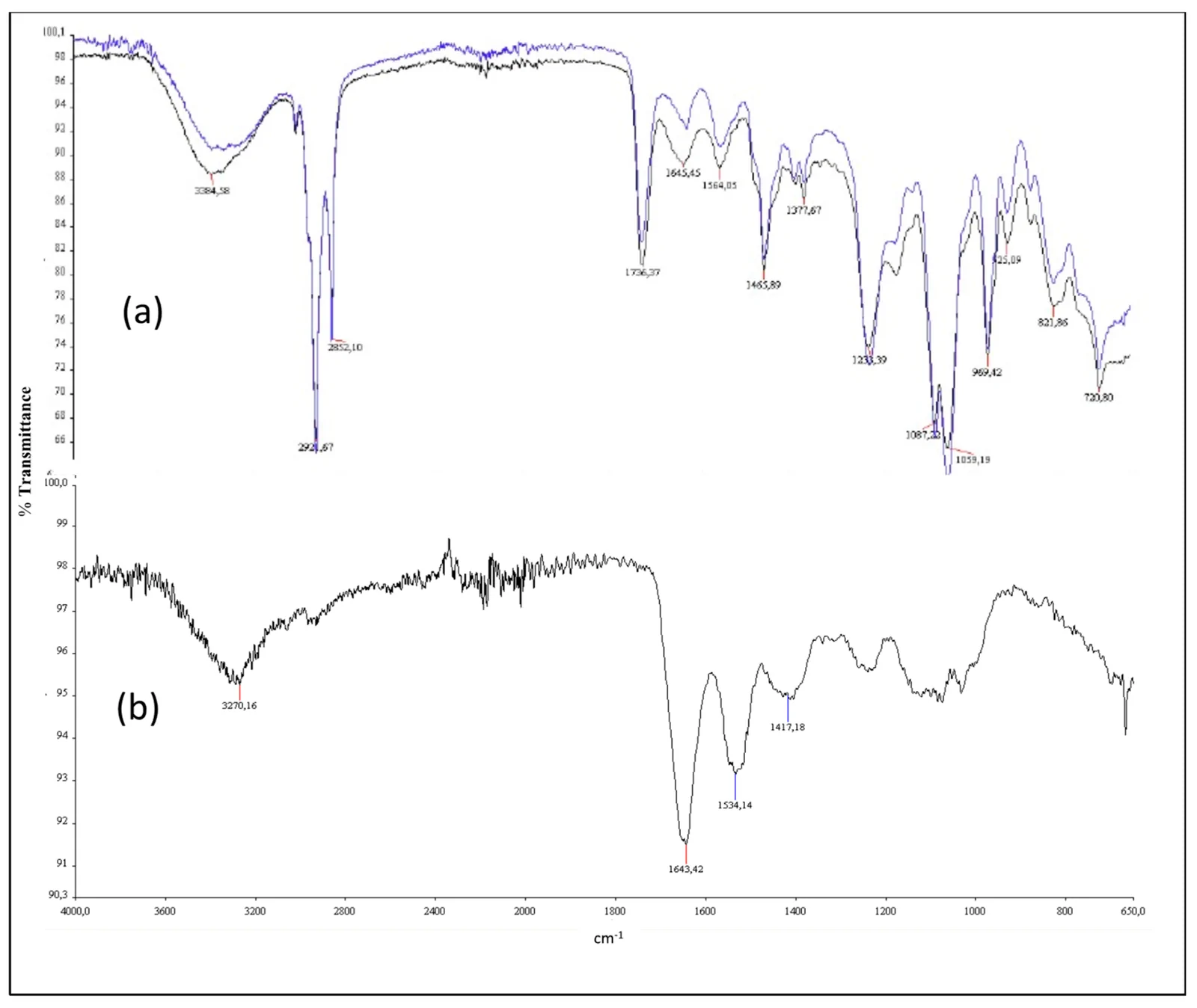
IR spectra of L ((**a**), blue), LHC ((**a**), black) and crude HC (**b**).

The IR spectra analyses reported in Figure 3 showed the liposome-related peaks of L (a) and LHC (b), that appeared in the wavenumber range between 3200–2800 and 1400–650 cm^−1^ compared to crude HC (c). As previously indicated in the Materials and Methods sections, the IR spectrum of HC presented: (i) the typical peaks at 3250 cm^−1^ (hydroxyl groups of hydrophilic amino acids); (ii) the aromatic overtone area between 3000 and 3200 cm^−1^ (due to aromatic amino acids); and (iii) proteins’ peaks at 1645 cm^−1^ and at 1540 cm^−1^, confirming the quality of the product. Concerning the IR evaluation of the liposomes’ formulations, we found an increased signal related to protein carbo-amino bonds at 3700–3500 cm^−1^ in LHC spectrum (a, black) as compared to that of L (a, blue), indicating a possible lipid–protein interaction. Moreover, a transmittance rate of 94% and 90% for L and LHC, respectively, is reported, therefore supporting the liposomes loading of HC. In addition, the IR spectra revealed that the loading of HC into liposomes using a brief ultrasonication process did not affect HC quality.

### 3.3. Biological Activity

**Figure 4:**
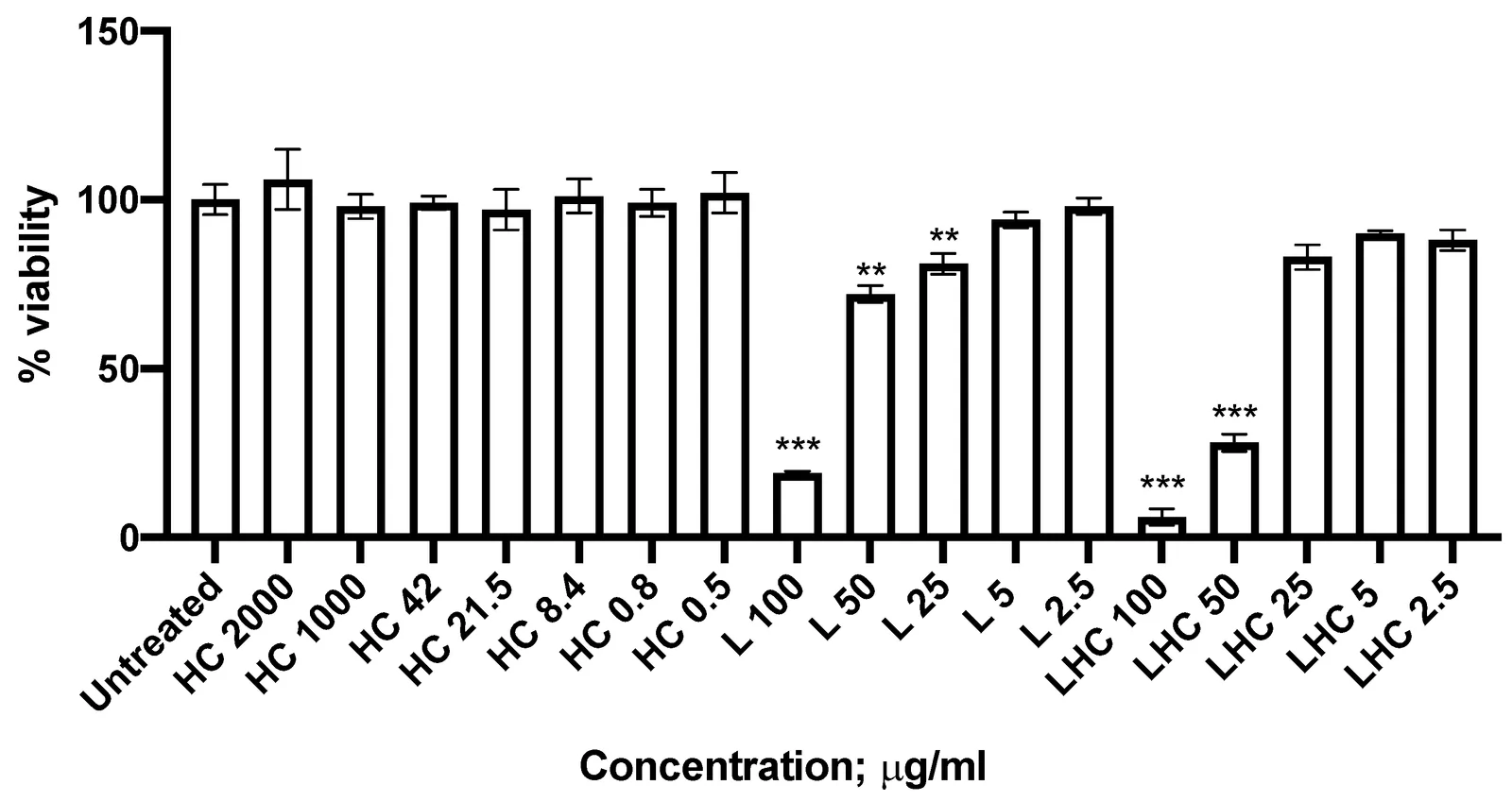
Cytotoxic activity on MRC-5 human fibroblast cells after 24 h treatment with HC, L and LHC. Evaluation was performed by means of MTT assay. Data are the mean % of three independent experiments ± SD. ** *p* < 0.01 and *** *p* < 0.001.

Liposome cell toxicity was evaluated on MRC-5 fibroblast cell line using scalar concentrations of L (empty liposomes) and LHC (loaded liposomes) (Table 2 and Figure 4).

HC was tested at the lowest concentrations that previously demonstrated a biological effect [9,10]. After 24 h of incubation at 37 °C, HC showed no cytotoxicity at both tested concentrations. On the contrary, L and LHC showed significant cytotoxicity at 100 µg/mL and 50 µg/mL (*p* < 0.0001; Student’s *t* test) but no cytotoxicity at lowest concentrations (Figure 4). Considering the concentration of 25 µM, we observe a decrease in cell viability after treatment with L, but not with LHC formulation. This difference may be attributed to protective function due to the content of HC present in the LHC preparation.

**Figure 5:**
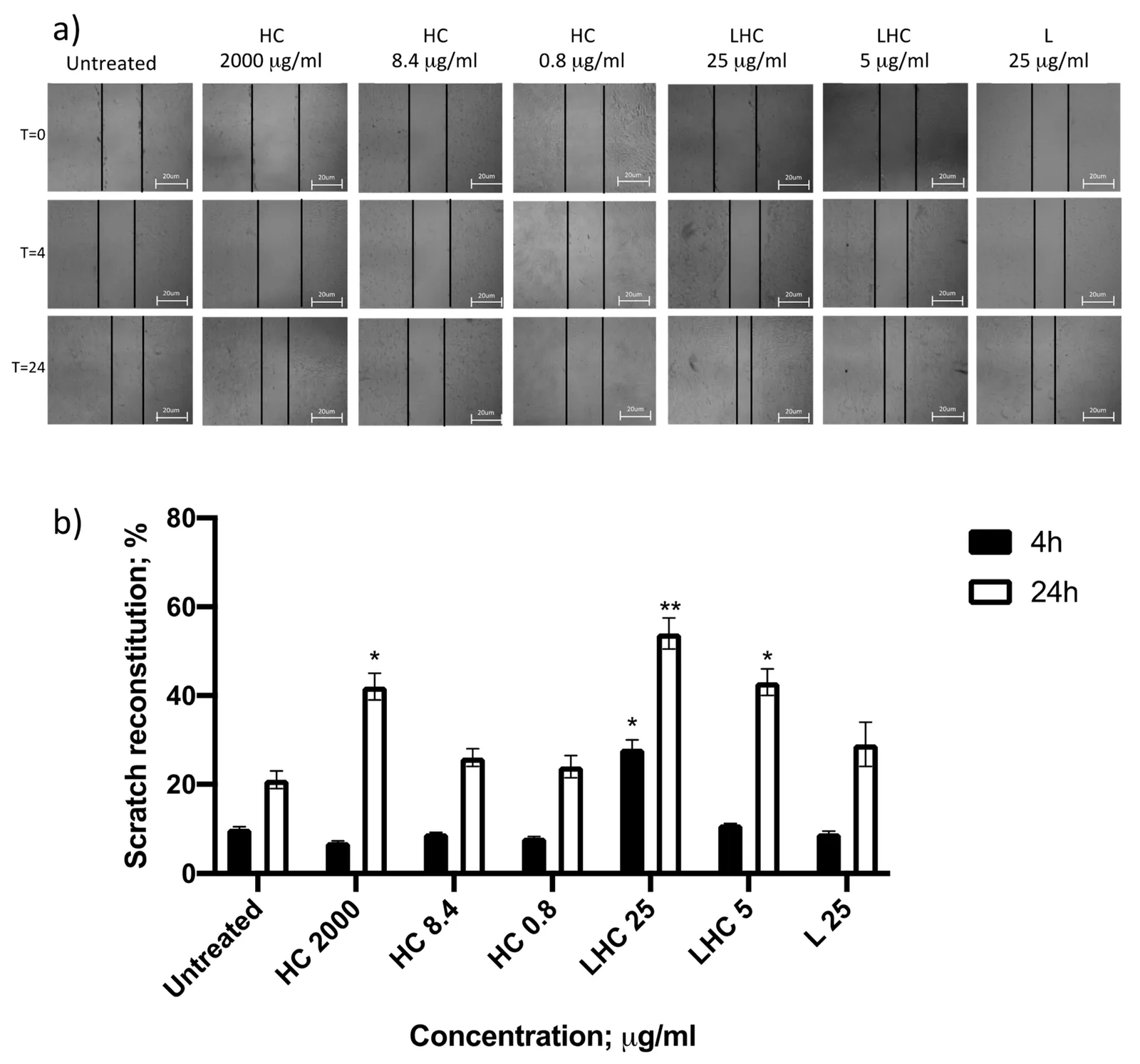
Evaluation of HC, LHC and L treatment on wound healing recovery in MRC-5 cells with images (**a**) and graphical representation (**b**) at the indicated time points. Data are the mean % of three independent experiments ± SD. * *p* < 0.05 and **p < 0.01.

On the basis of these results, we selected LHC at 25 µg/mL and 5 µg/mL for scratch-wound healing assay. As reported in Figure 5, the percentage of MRC-5 monolayer reconstitution after treatment with both LHC preparations was compared with the untreated control, empty liposomes (L) and crude HC at different concentrations, namely 2000 µg/mL (chosen as positive control) 8.4 µg/mL and 0.8 µg/mL, in order to provide a strong migratory effect without cytotoxicity, as previously reported [8]. The monolayer reconstitution was monitored at 4 h and 24 h after the scratch. 

We observed that after 4 h LHC 25 µg/mL induced a higher cell monolayer reconstitution (28%) as compared to the untreated (10%) and HC treated (7%) samples (*p* = 0.03 and *p* = 0.003, respectively; Student’s *t* test). The effect was more evident after 24 h, when the cell monolayer reconstitution in the presence of LHC was 54% in comparison with the 21.2% in untreated control and the 41.6% with HC treatment (*p* = 0.006 and *p* = NS, respectively; Student-*t* test) (Figure 5b). 

LHC 5 µg/mL reconstituted the wound similarly to the HC positive control (42.5%) at 4 h, while the wound reconstitution was more evident at 24 h in the presence of LHC (*p* = 0.012, Student’s *t* test) (Figure 5b). On the contrary, no significant results on cell scratch reconstitution were reported after treating with L 25 µg/mL at both time points analyzed.

In addition, the treatment with sole HC at the same concentration loaded on liposomes (8.4 and 0.8 µg/mL) showed no significant difference compared to the untreated control. Furthermore, the treatment with a physical mixture of L and crude HC (corresponding to the total amount of HC present in LHC 25 µg/mL, i.e., 20 µg/mL) resulted in a scratch reconstitution similar to that obtained from the untreated control (Figure S1). These data demonstrated that the best tissue repair is observed only when HC is loaded on liposomes (LHC), hence suggesting an efficient interaction of HC with the cell, possibly due to the different way in which HC is internalized within the cell [18,19,20]. 

## 4. Conclusions

As previously demonstrated by the evaluation of HelixComplex (HC) snail mucus’ chemical and biological characteristics, proteins and glycosaminoglycans are the main components allowing HC the ability to act as a mucosal barrier against external agents, thereby improving cell regeneration [9,10,16]. These biological effects are possible due to the interaction and synergistic activity of several small molecules or secondary metabolites characterizing this molecular complex.

Among these secondary metabolites, glycolic acid and allantoin are the most represented and exert the promotion of the cell growth and tissue repair processes. Unfortunately, in HC snail mucus the levels of both these molecules are extremely low [21]; therefore, it seems to be worth using biological carriers, such as liposomes, to enhance their bioavailability in reaching the highest concentrations inside the skin cells.

A preliminary study conducted in our laboratories indicated that cationic liposomes composed of PC, CH and OD showed a low cytotoxicity [7], are able to efficiently load HC (EE% > 93) and are stable up to 60 days. Therefore, LHC can be considered suitable to carry compounds into human cells [22].

In addition, LHC showed interesting effects on wound healing reconstitution, allowing higher biological effects to be reached without cytotoxicity already after 4 h of treatment, using very low amounts of HC (8.4 µg/mL vs. 2000 µg/mL of positive control). This evidence supports the use of liposomes to enhance HC efficacy, probably ascribable to endocytosis or membrane fusion processes, proposed as the main uptake mechanism in relation to large cationic liposomes [20,23]. Our data suggest that the HC internalization process is the basis of the improved efficacy observed for LHC in comparison with the crude HC, possibly because the substances within the crude HC cannot be completely internalized, or their internalization is slow. In fact, the use of cationic liposomes has been reported to be useful in increasing the intracellular distribution of the delivered substance, allowing it to reach the cell cytoplasm and eventually nucleoplasm [23], thus increasing their effects with respect to the treatment with the free form of the compound [24]. Indeed, it has been demonstrated that it was possible to appreciate a significant effect on wound healing even if the HC concentration within LHC was lower than that of crude HC. 

We are aware that further studies are needed to prove the effectiveness of this technological solution in improving snail mucus properties. An in vivo analysis will be necessary to confirm the efficacy of this delivery system and tissue distribution. However, our preliminary results are extremely promising and might also be proposed for other biological properties of HC.

## Figures and Tables

**Table 1:** Main physico-chemical characteristics of HelixComplex (HC) snail mucus.

Specification	Values	Unit of Measure
Aspect	Clear	-
Colour	Light Yellow	-
Odour	Odourless	-
Water Solubility	Soluble	-
Organic Solvents Solubility	Insoluble	-
pH	6–8	-
Density	1–1.1	g/mL
Dry Matter	2–3	%
Elements	250/350	mg/L
Heavy metals	According to Reg. 629/08	-
Proteins	200/350	mg/L
GAGs (Sulphated)	29/90	mg/L
Non-sulphated GAGs (Hyaluronic Acid)	70/80	mg/L
Glycolic Acid	<200	mg/L
Allantoin	<20	mg/L
Total Polyphenols	70/80	mg/L
Total Sugars	10/27	mg/L
Total Microbial Load	Absent	ufc/mL
Pesticides	According with REG 396/05 and subsequent updates	
Preservatives	Absent	-

**Table 2:** Relationship between concentrations of total lipids and HC (calculated on the basis of EE%) in the produced liposomes.

	Lipids (µg/mL)	HC (µg/mL)
Unloaded Liposomes (L)	100	/
	50	/
	25	/
	5	/
	2.5	/
Liposome-Carrying HC (LHC)	100	42
	50	21.5
	25	8.4
	5	0.8
	2.5	0.5

## Data Availability

The data presented in this study are available in Supplementary Material.

## Supplementary Materials

The following are available online, Figure S1: Evaluation of ultrasonicated HC 2000 μg/mL (a) and L + HC mixture (L 25 μg/mL + HC 20 μg/mL) (b) treatment on wound healing recovery in MRC-5 cells with images and graphical representation (c) at the indicated time points. Data are the mean % of three independent experiments ± SD. Figure S2: Evaluation of HC encapsulation efficacy (EE%) into LHC by HPLC analysis. (a) allantoin standard solution (retention time 4.6 min); (b) filtered LHC, the 4.6 min peak corresponds to allantoin while the others are related to the other HC components, such as glycolic acid; (c) spiked chromatogram of filtered LHC; (d) allantoin standard curve. AU: absorbance; uAs: unit of absorbance.
