# Correction: Alogna et al. Design of Liposomes Carrying HelixComplex Snail Mucus: Preliminary Studies. *Molecules* 2021, *26*, 4709

**DOI:** 10.3390/molecules31142528

**Published:** 2026-07-21

**Authors:** Andrea Alogna, Valentina Gentili, Claudio Trapella, Supandeep Singh Hallan, Maddalena Sguizzato, Giovanni Strazzabosco, Mercedes Fernández, Rita Cortesi, Roberta Rizzo, Daria Bortolotti

**Affiliations:** 1 Department of Chemical, Pharmaceutical and Agricultural Sciences, University of Ferrara, I-44121 Ferrara, Italy; gntvnt@unife.it (V.G.); trap@unife.it (C.T.); hllsnd@unife.it (S.S.H.); mercedes.fernandez@unife.it (M.F.);; 2 Laboratory for Technologies of Advanced Therapies (LTTA), University of Ferrara, 70 Eliporto Street, I-44121 Ferrara, Italy; 3 Biotechnology Interuniversity Consortium (C.I.B.), Ferrara Section, University of Ferrara, I-44121 Ferrara, Italy

In the original publication [1], the Conflicts of Interest statement of Andrea Alogna, Claudio Trapella and Roberta Rizzo was not included. The updated Conflicts of Interest should be as follows. The authors state that the scientific conclusions are unaffected. This correction was approved by the Academic Editor. The original publication has also been updated.

**Conflicts of Interest:** Authors Andrea Alogna, Claudio Trapella and Roberta Rizzo are founders of this HelixPharma. The remaining authors declare no conflicts of interest. HelixPharma has commercial interests related to HelixComplex. The company, HelixPharma, had no involvement in the design of the study, the collection, analysis, or interpretation of the data, the writing of the manuscript, or the decision to publish the results.
