# Supplementary material for: Design of Liposomes Carrying HelixComplex Snail Mucus: Preliminary Studies
Source: Molecules. 2021 Aug 4;26(16):4709. doi: 10.3390/molecules26164709 (PMC8399803; doi:10.3390/molecules26164709)
Supplement: Supplementary file 1 [file molecules-26-04709-s001.zip › molecules-1303781-supplementary.pdf]

Supplementary Materials

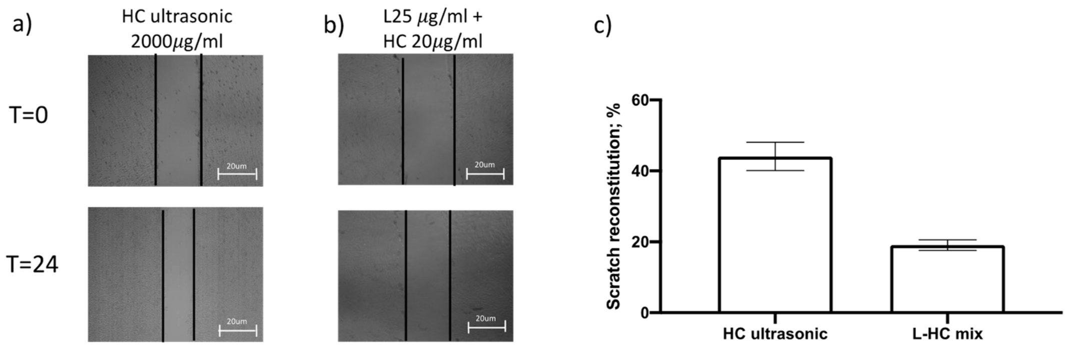

Figure S1. Evaluation of ultrasonicated HC 2000 μg/mL (**a**) and L + HC mixture (L 25 μg/mL + HC 20 μg/mL) (**b**) treatment on wound healing recovery in MRC-5 cells with images and graphical representation (**c**) at the indicated time points. Data are the mean % of three independent experiments ± SD.

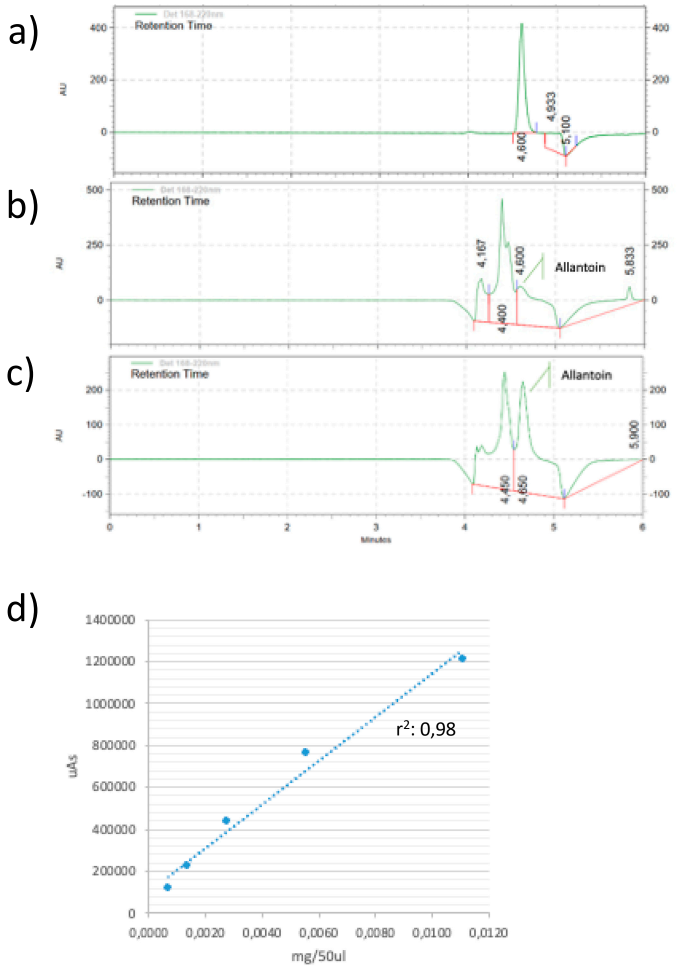

Figure S2. Evaluation of HC encapsulation efficacy (EE%) into LHC by HPLC analysis. (**a**) allantoin standard solution (retention time 4.6 min); (**b**) filtered LHC, the 4.6 min peak corresponds to allantoin while the others are related to the other HC components, such as glycolic acid; (**c**) spiked chromatogram of filtered LHC; (**d**) allantoin standard curve. AU: absorbance; uAs: unit of absorbance.
